# *Tabernaemontana catharinensis*: A Source of Indole Alkaloids With Potential Activity as Chemotherapeutic Agents

**DOI:** 10.1002/cbdv.202503532

**Published:** 2026-04-23

**Authors:** Ana Júlia Gomes Donada, Rafael Freitas Corá, Bianca Canci, Mariana Roesch‐Ely, Pauline Fagundes Rosales, Sidnei Moura

**Affiliations:** ^1^ LBIOP–Laboratory of Biotechnology of Natural and Synthetic Products Technology Department Biotechnology Institute University of Caxias do Sul Caxias do Sul Brazil; ^2^ Laboratory of Applied Toxicology and Bioproducts University of Caxias do Sul Caxias do Sul Brazil; ^3^ IFRS–Federal Institute of Education Science and Technology of Rio Grande Do Sul Campus Bento Gonçalves Brazil

**Keywords:** colorectal carcinoma, HCT116 cell, indole alkaloids, *Tabernaemontana* catharinensis

## Abstract

This study aimed to evaluate the antitumor activity of fractions rich in indole alkaloids extracted from the leaves of the species *Tabernaemontana catharinensis*. The chemical composition was analyzed using high‐resolution mass spectrometry (HRMS), which identified nine known indole alkaloids, including voacangine, voacangine hydroxyindolenine, ibogamine, and affinisine. In the subsequent step, both the extract and the fractions were tested against the human colorectal carcinoma cell line HCT116. Cytotoxicity assays showed that the total alkaloid fraction (AF) exhibited selectivity against this tumor cell line (IC_50_ 28.49 µg mL^−1^), while preserving the viability of the non‐tumor MRC5 cell line (IC_50_ 60.35 µg mL^−1^) after 24 h of treatment. Additionally, it exhibited moderate cytotoxicity in the brine shrimp (*Artemia salina*) assay (LC_50_ 367.33 ± 2.45 µg mL^−1^). The results obtained in this study suggest that the alkaloids present in the fractions and subfractions synergistically have potential for the development of selective chemotherapeutic agents for colorectal carcinoma.

## Graphical abstract

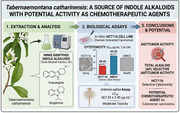

## 1 Introduction

Historically, plants have played a significant role in the search for bioactive compounds to treat diseases—a pursuit that has been greatly advanced by improvements in chemical characterization techniques. In this context, the bioactivity of plants arises from secondary metabolites, which are responsible for their adaptation [1]. These metabolites can be broadly classified into major groups: terpenes, phenolics, and nitrogen‐containing compounds, including alkaloids [2]. Alkaloids, in turn, can be further categorized based on their nitrogen‐containing heterocyclic core structures into classes, such as pyrroline, pyridine, and piperidine, tropanes, pyrrolizidine, quinoline and isoquinoline, terpenoids, steroids, and indole alkaloids [3, 4]. Among these, indole alkaloids are defined as tryptophan‐derived compounds that feature a nitrogen atom within a fused pyrrole‐benzene ring structure [5, 6].

Plant secondary metabolites represent a valuable source of potential anticancer agents. Continued research and development in this area holds promises for improving cancer treatment outcomes. One prominent example is Taxol (paclitaxel), which is isolated from the bark of the Pacific yew tree (*Taxus brevifolia*) and is widely used as a chemotherapy drug for various cancers [7]. Regarding indole alkaloids, vinblastine and vincristine, derived from the Madagascar periwinkle plant (*Catharanthus roseus*), are used to treat leukemias and lymphomas [8]. Colorectal cancer is one of the most prevalent malignancies worldwide and represents a significant public health concern, highlighting the need for the discovery of new bioactive compounds with potential therapeutic applications [9]. The species *Tabernaemontana catharinensis* A.DC, from the *Apocynaceae* family, is widely recognized for its use in traditional medicine [10]. *T. catharinensis* is commonly referred to as “cobrina,” “leiteira,” “jasmim,” or “jasmim‐catavento” [11] and is an arboreal species found primarily in South America, particularly in Brazil [12]. Its diverse biological activities—including anti‐inflammatory [13], leishmanicidal [14], antinociceptive [15], anticholinesterase [14], antioxidant [15, 16], antimycobacterial [16], and anticancer effects [17]—are mainly attributed to its content of indole alkaloids. In a historical investigation of active indole alkaloids from this species, six compounds were isolated, including affinisine. This compound has previously been isolated and evaluated against cancer cell lines, such as A549, A375, and VERO, presenting IC_50_ values of 58.67 ± 6.14 and 11.73 ± 36.25 µg mL^−1^, respectively, with observed selectivity [18].

Additionally, extracts of *T. catharinensis* have been evaluated for their ability to inhibit acetylcholinesterase, showing an IC_50_ value of 261.55 ± 9.1 µg mL^−1^ [14]. Furthermore, fractions obtained from this species have demonstrated antinociceptive activity in experimental pain models without presenting relevant toxicological effects [19]. Reviews on *Apocynaceae* have further highlighted the therapeutic potential of species from this family, particularly due to their rich content of indole alkaloids [6]. However, despite the diversity of indole alkaloids reported for this species, studies investigating their cytotoxic potential against colorectal cancer cell lines remain limited. Based on this context, the present study aims to obtain extracts and fractions enriched in alkaloid compounds from *T. catharinensis*, which were subsequently characterized using high‐resolution mass spectrometry (HRMS). The cytotoxic potential of the extracts will first be evaluated using the *Artemia salina* lethality assay, a nontarget toxicity model widely employed as a preliminary indicator of general bioactivity. This will be followed by an antitumoral assay against HCT116 (human colorectal carcinoma) cells, with cytotoxic selectivity assessed through comparative analysis using MRC5 (normal human fibroblast) cells. Thus, this study contributes to the ongoing search for novel chemotherapeutic prototypes based on natural products.

## 2 Results and Discussion

Secondary metabolites produced by plants serve various purposes, such as defense against herbivores and attraction of pollinators, and exhibit significant biological activity. Alkaloids have been highlighted in cancer treatment [20], culminating in the discovery of drugs still in use today, such as Taxol [7], vinblastine, and vincristine [8]. In this context, we emphasize indole alkaloids as potential chemotherapeutics, including affinisine, which shows promise against colon cancer and glioblastoma cell lines in acoor [21]. Our research group has a history of investigating these compounds [6, 14, 22], which has led us to believe that they may be on the horizon of new chemotherapy drugs.

In this study, we performed ultrasound‐assisted extraction using a polar solvent. Sonication has proven effective for the extraction of polar plant compounds, such as alkaloids. Alkaloid extraction yields reported in the literature vary widely depending on the plant species and extraction method. For instance, neferine obtained from *Nelumbo nucifera* presented a yield of 0.86% (w/w) [23], while sonication‐assisted extraction of total alkaloids from *Annona muricata* reached 26.38% [24]. Ultrasound optimizes the extraction of compounds from plant cells based on the phenomenon of cavitation [25].

Alkaloids are primarily basic due to the presence of nitrogen as a heteroatom, making them preferentially extracted with organic solvents through pH adjustment. In the next step, we used a combination of acid–base extraction with ethyl acetate and dichloromethane, yielding 0.94% (m/m) of the alkaloid fraction (AF). For this species, AF yields of 0.75% and 0.1% have previously been reported using the same extraction approach [18, 26].

**FIGURE 1:**
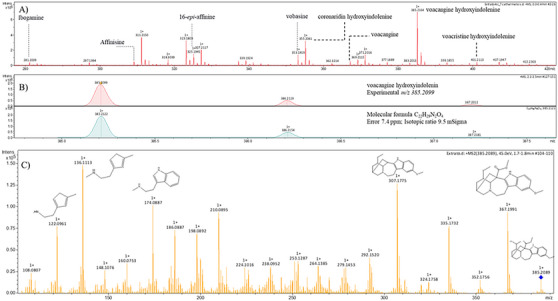
(A) Full mass spectrum obtained by HRMS in positive ESI mode showing the major ions detected in the alkaloid fraction. (B) Expanded view of the spectrum used for exact mass and isotopic pattern confirmation of the ion at *m/z* 385.2099 corresponding to voacangine hydroxyindolenine. (C) MS/MS fragmentation spectrum of the precursor ion at *m/z* 385 assigned to voacangine hydroxyindolenine.

HRMS has been widely used for the characterization of plant secondary metabolites in extracts, such as flavonoids, phenolics, terpenoids, coumarins, saponins, and alkaloids [27]. Tools, such as exact *m/z*, isotopic ratio, and fragmentation pathways, enable compound confirmation. For example, isobaric indole alkaloids from *T. catharinensis* have previously been characterized using these approaches [14, 22]. In this study, we identified nine different compounds in the AF: ibogamine (*m/z* 281.1963), affinisine (*m/z* 309.1933), 16‐epi‐affinin (*m/z* 325.1903), vobasin (*m/z* 353.1853), coronaridine hydroxyindolenine (*m/z* 355.2006), voacangine (*m/z* 367.1948), voacangine hydroxyindolenine (*m/z* 385.2093), *vocacristine* hydroxyindolenine (*m/z* 401.1987), and 12‐methoxy‐n‐methylvoachalothin (*m/z* 411.2265), Table 1. Among these, the alkaloid voacangine hydroxyindolenine stands out, as it was not observed in other studies conducted by our group. The accurate mass, isotopic pattern, and diagnostic fragmentation pattern obtained by MS/MS experiments are shown in Figure 1 (panels A–C), supporting the structural annotation of this compound. This discrepancy may be related to differences in extraction methods, considering the acid–base extraction [18], which used different solvents in this extraction step, and the Soxhlet extraction [14].

**TABLE 1:** The main compounds identified in the AF were characterized by ESI–HRMS using exact *m/z* values, isotopic ratio fitting, and fragmentation pathways.

Compound	Precursor ion	Fragmentation (%)	Difference (ppm)	Isotopic ratio (mSig)	Identification	References
01	281.1963	170.0950 (38), 158.0942 (39), 144.0810 (87), 138.1272 (52), 122.0963 (100).	3.0	12.8	Ibogamine	[12, 34]
02	309.1933	122.0963 (100.0), 138.0915 (42.95), 174.0905 (62.47), 200.1055 (32).	3.7	11.0	Affinisine	[12, 33, 34]
03	325.1903	309.1960 (35), 234.1260 (60), 180.0980 (33), 152.1059 (100), 135.1105 (63), 122.0954 (37), 108.0818 (11).	1.9	6.2	16‐*epi*‐affinine	[20, 34]
04	353.1853	323.1734 (29), 180.1016 (20), 144.0803 (98), 138.1225 (34), 122.0961 (31), 108.0814 (20).	2.9	6.2	Vobasine	[20, 31]
05	355.2006	180.998 (19), 160.0745 (34), 144.0806 (99), 138.1265 (32), 122.0957 (32), 108.0807 (19).	2.4	12.9	Coronaridin hydroxyindolenine	[3, 20, 31]
06	367.1948	335.1711 (20), 307.1500 (32), 180.1101 (100), 174.0886 (95), 160.0753 (85), 144.0801 (79), 136.1104 (56), 122.0963 (91).	5.1	5.9	Voacangine	[4, 20, 31]
07	385.2093	367.2017 (48), 335.1760 (31), 307.1785 (26), 174.0901 (55.28), 136.1111 (100), 122.0957 (51), 108.0808 (10).	2.9	7.4	Voacangine hydroxyindolenine	[5]
08	401.1987	339.1694 (56), 307.1414 (20), 269.1280 (80), 185.0693 (78), 174.0865 (43), 160.0736 (37), 144.0809 (100), 108.0808 (53), 99.0806 (25).	5.5	12.1	Voacristine hydroxyindolenine	[5, 20]
09	411.2265	200.1085 (75), 180.0982 (82), 156.1346 (12), 138.0846 (10), 122.0940 (29).	4.7	5.4	12‐methoxy‐n‐methylchalotin	[20, 31]

The search for active compounds in plants is challenging. However, a single compound can serve as an ideal prototype for semi‐synthetic modification or synthesis, as well as for in silico analysis to generate new active compounds. According to literature reports, affinisine shows cytotoxic activity against the U251 (glioblastoma) and HT29 (colorectal adenocarcinoma) cell lines, presenting IC_50_ values of 9.5and 9.3 µM, respectively. In the present study, we generated six fractions (named F1‐F6), three of which were rich in voacangine, while the others contained different indole alkaloids as their main components, as detailed in Table 2.

**TABLE 2:** Predominant alkaloids identified by ESI–HRMS in each sub‐fraction.

Fraction	Main alkaloids	Precursor ion	Retention time (min)	Molecular formula
F1	Ibogamine	281.1376	2.95	C_19_H_24_N_2_
F1	Affinisine	309.1909	4.55	C_20_H_24_N_2_O
F2	16‐*epi*‐affinine	325.1903	30.81	C_20_H_25_N_2_O_2_
F3	Vobasine	353.1879	40.01	C_21_H_24_N_2_O_3_
F4	Voacangine	367.2000	44.83	C_22_H_27_N_2_O_3_
F5	Voacangine	367.1973	57.33	C_22_H_27_N_2_O_3_
F6	Voacangine hydroxyindolenine	353.2652	66.09	C_21_H_24_N_2_O_3_

To understand the toxic activity of the crude extract, an initial screening with the AF against *A. salina* was performed. This microcrustacean is commonly used to assess toxicity due to its quick and low‐cost testing method, which correlates well with cytotoxic effects [28, 29]. As a result, the LC_50_ was determined to be 367.33 ± 2.45 µg mL^−1^, with values between 100 and 500 µg mL^−1^ considered moderately toxic [30]. Literature data indicate LC_50_ values of 629.35 ± 0.12 µg mL^−1^ for leaves and 504.18 ± 0.15 µg mL^−1^ for branches of hydroalcoholic extracts from *T. catharinensis* [28]. In comparison, an LC_50_ value of 883.34 µg mL^−1^ has been reported for *Tabernaemontana sphaerocarpa* [31].

According to the Pan American Health Organization (2024), it is estimated that in 2022, an average of 9.7 million deaths occurred due to cancer, with around 20 million new cases reported [32]. Among these figures, colorectal cancer ranks third, with 1.9 million cases. In Brazil, in 2022, an average of 28.800 deaths were attributed to colorectal cancer, accounting for 41% of the total in Latin America [33]. The projection for the 3‐year period from 2023 to 2025 estimates that there will be 46,000 new cases of colorectal cancer in the country [34].

**FIGURE 2:**
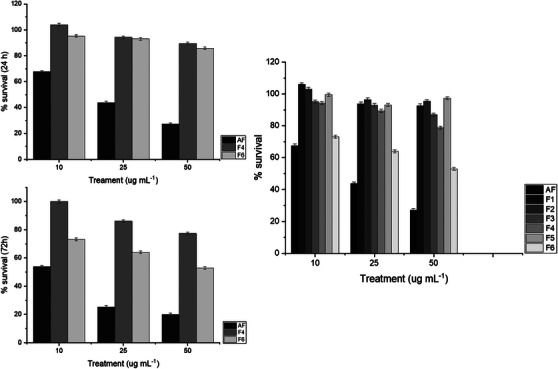
Cell viability of HCT116 (human colorectal carcinoma) cells treated with the total alkaloid fraction (AF) and selected subfractions (F4 and F6), obtained from chromatographic fractionation (F1–F6), at increasing concentrations (10–50 µg mL^−^^1^). (A) Cell viability after 24 h of treatment; (B) cell viability after 72 h of treatment. Viability was determined by the MTT assay and expressed as a percentage relative to untreated control cells (100% viability). Data are presented as mean ± standard deviation (*n* = 3).

One way to identify selective compounds as potential chemotherapeutic agents is to test them on immortalized cell lines of specific cancers. In the case of colorectal cancer, the HCT116 cell line is widely used in activity tests. Previous studies have reported that the indole alkaloids voacangine and coronaridine hydroxyindolenine, isolated from *Tabernaemontana divaricata*, exhibited cytotoxic activity against the HCT116 cell line, with an IC_50_ value of 40 µM [35]. In our study, we obtained an IC_50_ of 28.5 µg mL^−1^ for the AF after 24 h (Table 2). Compared to normal cells, such as MRC5 (human fibroblasts), we observed a tendency for selectivity, as shown in Figure 2.

On the other hand, the fractions enriched with other alkaloids did not perform well (Table 3), suggesting that the superior effect of the extract is due to a synergistic interaction among the chemical compounds present. This observation is consistent with previous findings indicating that crude extracts from *Tabernaemontana* species may exhibit greater activity than their isolated compounds [36].

**TABLE 3:** IC_50_ values (µg mL^−1^) of the total AF and its subfractions (F1–F6) against HCT116 (human colorectal carcinoma) and MRC5 (non‐tumor human fibroblast) cell lines after 24 and 72 h of treatment.

IC_50_ values (µg mL^−1^)										
24 h								72 h		
	AF	F1	F2	F3	F4	F5	F6	AF	F4	F6
HCT116	28.49 ± 2.66	121.14 ± 13.13	160.18 ± 34.9	224.11 ± 35.05	228.51 ± 4.98	207.52 ± 31.73	161.77 ± 9.85	20.86 ± 0.16	121.56 ± 8.26	51.43 ± 4.05
MRC5	60.35 ± 3.25	—	—	—	—	—	—	—	—	—

IC_50_ values were determined by nonlinear regression analysis of dose–response curves using a four‐parameter logistic model. Data are expressed as mean ± standard deviation (*n* = 3).

—not evaluated in this study.

**FIGURE 3:**
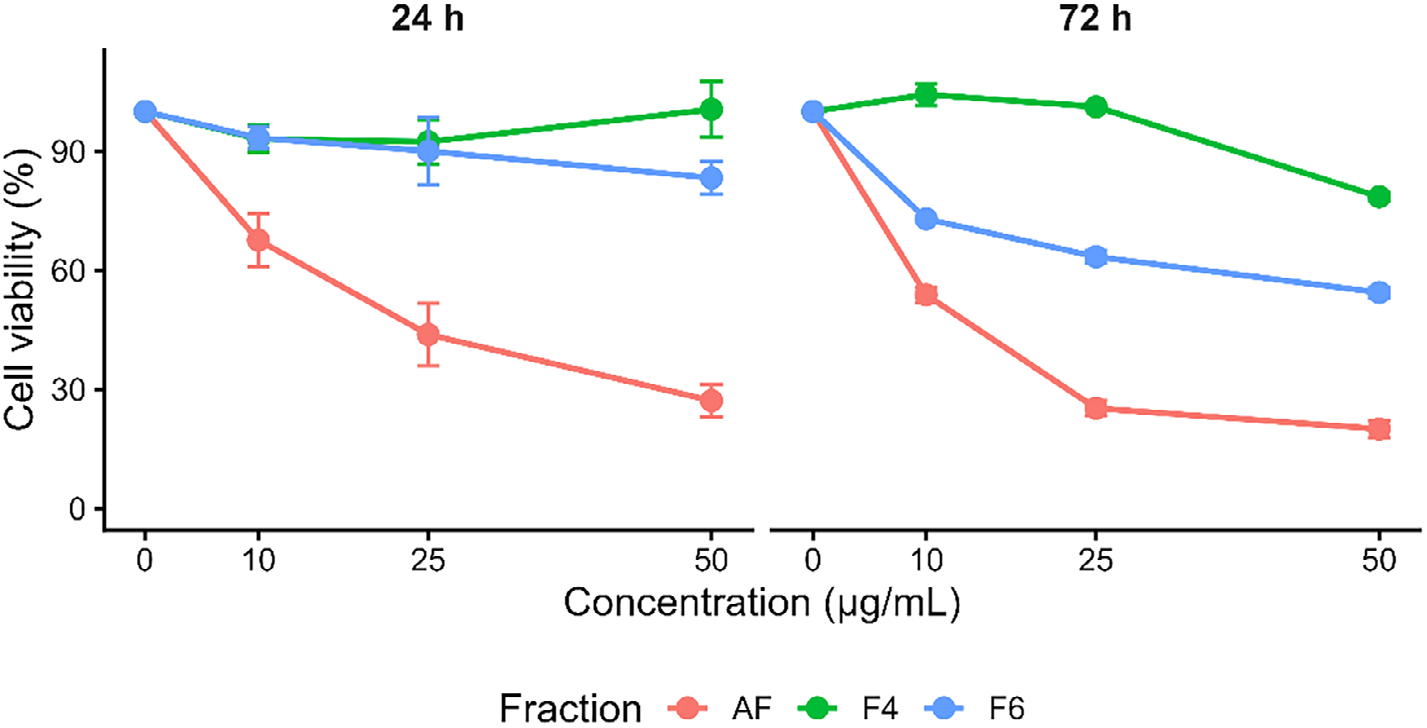
Dose–response curves of the total alkaloid fraction (AF) and subfractions (F4 and F6) evaluated against HCT116 (human colorectal carcinoma) cells after 24 and 72 h of treatment. Cell viability (%) was determined using the MTT assay and expressed relative to untreated control cells (100% viability). Cells were exposed to increasing concentrations (10, 25, and 50 µg mL^−1^), and data are presented as mean ± standard deviation (*n* = 3). Statistical differences were evaluated by three‐way ANOVA followed by Tukey's post hoc test (*p < 0.05, **p < 0.01, ***p < 0.001).

To further validate the cytotoxic effects observed, a statistical analysis was performed using three‐way ANOVA followed by Tukey's post hoc test. This approach allowed the identification of significant differences among treatments, concentrations, and exposure times. The dose–response profiles of the AF and selected subfractions (F4 and F6) are presented in Figure 3. A clear concentration‐dependent reduction in cell viability was observed for AF, particularly after 72 h of treatment, confirming its higher cytotoxic potential. In contrast, F4 showed no evident cytotoxic effect, while F6 exhibited a moderate reduction in viability, especially at higher concentrations and longer exposure times.

To better visualize these differences, Table 4 summarizes the cell viability values along with the statistical groupings based on Tukey's test. For AF, significant differences were observed between concentrations, reinforcing a dose‐dependent effect. On the other hand, F4 did not show statistically significant differences among concentrations, indicating low or absent cytotoxic activity. Fraction F6 presented intermediate behavior, with significant reductions at higher concentrations, particularly after 72 h. These results corroborate the IC_50_ data and further support the selective cytotoxicity of AF against HCT116 cells, suggesting that the biological activity may be associated with the combined effect of its alkaloid constituents.

**TABLE 4:** Effects of the total alkaloid fraction (AF) and subfractions (F4 and F6) on the viability of HCT116 (human colorectal carcinoma) cells after 24 and 72 h of treatment. Cell viability (%) was determined by the MTT assay after exposure to increasing concentrations (10, 25, and 50 µg mL^−^^1^), and IC_50_ values were calculated to estimate cytotoxic potency.

Compound	Time of treatment (h)	Viability 10 µg/mL (%)	Viability 25 µg/mL (%)	Viability 50 µg/mL (%)	IC_50_ (µg/mL)
AF	24	67.6 ± 6.7^a^	43.8 ± 7.8^b^	27.1 ± 4.2^c^	28.5 ± 2.66
	72	53.8 ± 1.9^a^	25.2 ± 1.9^b^	20.0 ± 2.1^b^	20.9 ± 0.16
F4	24	93.1 ± 3.5^a^	92.4 ± 5.6^a^	100.5 ± 7.0^a^	>200
	72	104.3 ± 2.7^a^	101.2 ± 0.6^a^	78.5 ± 1.0^b^	>100
F6	24	93.3 ± 2.7^a^	90.0 ± 8.5^a^	83.3 ± 4.2^a^	161.77 ± 9.85
	72	72.9 ± 0.5^a^	63.4 ± 1.6^b^	54.4 ± 1.4^c^	51.43 ± 4.05

Data are expressed as mean ± standard deviation (*n* = 3). Statistical analysis was performed using three‐way ANOVA followed by Tukey's post hoc test. Different lowercase letters (*a*–*c*) within the same row indicate statistically significant differences among concentrations for the same compound and exposure time (*p* < 0.05), while identical letters indicate no significant difference between groups.

## 3 Conclusions

In this study, we tested an alkaloid‐enriched extract that demonstrated good activity and selectivity, although the results for the enriched subfractions fell below expectations. This suggests that synergistic interactions between the compounds may be a significant factor. The results indicated that the alkaloids present in the total fraction (AF) and in fraction F6 exhibited strong selectivity, particularly highlighting their antitumor activity against the HCT116 cell line while maintaining selectivity against the non‐tumor MRC5 cell line. Therefore, the indole alkaloids in these fractions can be considered promising antitumor agents based on the cytotoxic activities observed in the conducted tests. We continue our search for promising prototypes for cancer treatments, with this class of compounds showing potential as new therapeutic agents.

## 4 Experimental Section

### 4.1 Chemicals

The reagents and solvents, such as ethanol (EtOH), used in the chemical process were purchased from Moderna (São Paulo, Brazil) and Merck (Darmstadt, Germany) with a purity higher than 99.5%. For the *A. salina* assay, the positive control potassium dichromate (K_2_Cr_2_O_7_) was obtained from Labsynth Produtos para Laboratórios (São Paulo, Brazil). For cell tests, Dulbecco's Modified Eagle Medium–High Glucose (DMEM), heat‐inactivated fetal bovine serum (FBS), trypsin–EDTA, 3‐(4,5‐dimethylthiazol‐2‐yl)‐2,5‐diphenyl‐tetrazolium bromide (MTT), and dimethyl sulfoxide (DMSO) were purchased from Sigma–Aldrich (St. Louis, MO, USA) with a purity higher than 99.9%.

### 4.2 Plant Material

The leaves of *T. catharinensis* (approximately 20 kg) were collected in Ijuí, Rio Grande do Sul, Brazil, in January 2023 (28°26′06″ S, 53°56′05″ W). The collection was identified by Felipe Gonzati and deposited in the Caxias do Sul University Herbarium (exsiccate HUCS 51585) in Caxias do Sul, Rio Grande do Sul, Brazil.

### 4.3 Extraction and Fractionation

The plant material was dried in a greenhouse with forced air circulation at 30°C for 96 h and then ground using a knife mill. In the subsequent step, approximately 4 kg of the material was extracted using ultrasound with ethanol (EtOH) at a ratio of 10 mL per gram, at 40% amplitude, and 500 W power for 30 min. The solution was then filtered, and the solvent was evaporated using a rotary evaporator. Following this step, the crude extract underwent an acid–base extraction procedure, adapted from a previously described method [18]. Specifically, around 591 g of ethanolic crude extract, obtained by ultrasound‐assisted extraction, was diluted with 100 mL of 1% hydrochloric acid (HCl) and extracted with ethyl acetate (EtOAc) in three 100 mL portions. The aqueous phase was basified to pH 11 using 1 M sodium hydroxide (NaOH) and re‐extracted with dichloromethane (CH_2_Cl_2_) in three 100 mL portions. The solvent from the organic phase was evaporated, yielding an AF (named AF). The AF was then fractionated using preparative liquid chromatography on a Shimadzu Prominence system equipped with a Supelco Analytical C18 column (25 cm × 10 mm, 5 µm), a Shimadzu FRC10A fraction collector, and a PDA detector (SPD‐M20A) operating at wavelengths between 250 and 450 nm at a frequency of 1562 Hz. The mobile phase operated in gradient mode and consisted of a binary solvent system comprising 0.1% ammonium hydroxide (A) and methanol (B), delivered at a flow rate of 2 mL min^−1^: from 0 to 5 min, 40% B; from 5.01 to 60 min, 80% B; at 70 min, 80% B; at 75 min, 40% B, and then maintained for 5 min. The injection volume was 10 µL, and a total of 2 g of the AF was processed under these chromatographic conditions. In total, six enriched fractions, identified from F1 to F6, were collected after 80 min of HPLC running, concentrated using a rotary evaporator, and analyzed by HRMS.

### 4.4 Chemical Characterization by HRMS

The fractions were diluted in methanol to a final concentration of 10 µg mL^−1^ and individually injected using a syringe pump (Harvard Apparatus, Hamilton, Reno, Nevada) at a flow rate of 180 µL min^−1^ into a mass spectrometer (Bruker Scientific, Billerica, USA), model microTOF‐Q II, which employs electrospray ionization (ESI) and a quadrupole‐time‐of‐flight (Q‐TOF) mass filter. For compound identification, an ion acquisition mass range of *m/z* 200–800 was utilized, with a scanning rate of two scans per second. The capillary entrance voltage was set to +4500 V, the nebulizer gas pressure was maintained at 0.5 bar, the temperature was adjusted to 180°C, and the drying gas flow rate was set to 4 L min^−1^, resulting in a resolution of approximately 25,000 (FWHM) at *m/z* 200. The voltages for the end plate offset and collision were established at −500 V and −1 V, respectively. Targeted MS/MS experiments were performed by selecting specific precursor ions and applying collision‐induced dissociation at different collision energies to obtain diagnostic fragmentation spectra. Diagnostic ions were identified by comparing their *m/z* values, isotopic ratios, and fragmentation pathways with those of previously identified compounds. Data acquisition and processing were conducted using OTOF Control and DataAnalysis software from Bruker Scientific.

### 4.5 Determination of Cytotoxic Activity

#### 4.5.1 *A. salina* Assay

A glass tank was filled with artificial seawater (salt mixture: 36.5 g L^−1^), and 50 mg of brine shrimp eggs (acquired from an aquarium supply store) were added. The tank was incubated for 48 h under artificial light until the nauplius stage was reached [37]. The alkaloid extract and the positive control (K_2_Cr_2_O_7_) were prepared in triplicate at concentrations of 1000, 500, 250, 125, and 0 µg mL^−1^. These solutions were transferred to wells containing 800 µL of marine solution and 10 nauplius larvae each.

#### 4.5.2 Antitumoral Assay

The HCT116 (human colorectal carcinoma cell line, RRID:CVCL_0291) and MRC5 (human lung fibroblast cell line, RRID:CVCL_0440) cell lines, obtained from the Banco de Células do Rio de Janeiro (BCRJ, Brazil), were cultured in DMEM‐supplemented media with antibiotics (1% penicillin and streptomycin) and 10% FBS (Gibco BRL/Life Technologies, Carlsbad, CA, USA) at 5% CO_2_ and 37°C. Following the protocol [38], the cell lines were added to 96‐well flat‐bottom microplates at a density of 8 × 10^4^ cell mL^−1^ for the HCT116 and 1 × 10^5^ cell mL^−1^ for the MRC5, with 10% FBS in DMEM. After cell allocation, serial dilutions of alkaloid extract and fractions with increasing concentrations (10, 25, and 50 µg mL^−1^) at 37°C in the growing medium were added to the cells for 24 and 72 h. Ethanol 2.5% (v/v) was used as a negative control. In accordance with the protocol, the MTT solution was removed after 2 h of incubation, and the formazan crystals were dissolved by adding DMSO (100 µL), followed by shaker for 30 min, protected from light. The absorption was determined at 570 nm (SpectraMax 190, Molecular Devices, San Jose, CA, USA), and the results were determined as percentage viability of the negative control.

### 4.6 Statistical Analysis

The LC_50_ values from the *A. salina* assay were calculated using probit analysis based on the best‐fit line obtained through linear regression [39], implemented in R (version 4.5.2; R Foundation for Statistical Computing, Vienna, Austria) via RStudio (version 2026.01.0). This procedure yielded estimates with 95% confidence intervals. For the cytotoxicity assays, half‐maximal inhibitory concentrations were obtained by nonlinear regression of dose–response curves using the drc package in R. Cell viability percentages were plotted against the logarithm of the concentrations and fitted with a four‐parameter logistic model. These values were derived from three independent experiments and reported with 95% confidence intervals. Statistical significance among treatments was evaluated by three‐way analysis of variance (ANOVA), considering compound, concentration, and treatment time as factors, followed by Tukey's post hoc test for multiple comparisons. Results were expressed as mean ± standard deviation (SD), and differences were deemed statistically significant at *p* < 0.05.

## Author Contributions

**Ana Júlia Gomes Donada**: conceptualization, methodology, formal analysis, investigation, data curation, writing – original draft, writing – review and editing. **Rafael Freitas Corá**: methodology, investigation, writing – review and editing. **Bianca Canci**: methodology (MTT assay), investigation. **Mariana Roesch‐Ely**: supervision, writing – review and editing. **Sidnei Moura**: formal analysis, supervision, validation, visualization, writing – review and editing.

## Funding

The authors declare financial support was received for the research, authorship, and/or publication of this article. Fundação de Amparo a Pesquisa do Estado do Rio Grande do Sul – FAPERGS (Edital 09/2023 Programa Pesquisador Gaúcho – PQG, Termo De Outorga: 24/2551‐0001302‐4). Conselho Nacional de Desenvolvimento Científico e Tecnológico – CNPq (Edital Universal – 2025) and for Coordenação de Aperfeiçoamento de Pessoal de Nível Superior – CAPES.

## Conflicts of Interest

The authors declare no conflicts of interest.

## Data Availability

The data that support the findings of this study are available from the corresponding author upon reasonable request.
